# A case of metastatic neuroendocrine disease and cholecystitis: surgical remedy and management of carcinoid crisis

**DOI:** 10.1093/jscr/rjab543

**Published:** 2021-12-11

**Authors:** Phillip J Whiley, Janaka Balasooriya, Rudyard J Wake

**Affiliations:** Australian National University Medical School, Canberra, ACT, Australia; Division of General Surgery, The Canberra Hospital, ACT, Australia; Division of General Surgery, The Canberra Hospital, ACT, Australia; Division of General Surgery, The Canberra Hospital, ACT, Australia

## Abstract

The report presents a case of a 70-year-old male with a known mesenteric neuroendocrine tumour and metastases to the liver diagnosed with acute cholecystitis. During surgery, the patient developed a carcinoid crisis with mixed distributive and cardiogenic shock involving systemic vasodilation and arrhythmia. During surgical procedures, carcinoid crisis can be precipitated by tumours that secrete a pathological shower of vasoactive mediators. Somatostatin analogues are utilized to control carcinoid syndrome and are routinely used peri-operatively. However, no standard infusion regimen exists. The case raises the suggestion that metastatic liver neuroendocrine disease may confound the diagnosis of cholecystitis, complicates the management of acute surgical presentations and highlights the need for agreement on octreotide therapy for surgical patients with carcinoid tumours.

## INTRODUCTION

Neuroendocrine neoplasms (NENs) are derived from enterochromaffin cells mainly present in the gastrointestinal tract. NENs are subdivided into the poorly differentiated neuroendocrine carcinomas (NECs) and well-differentiated neuroendocrine tumours (NETs) and approximately 20% of patients with NENs experience carcinoid syndrome [1]. Carcinoid crisis is a severe form of carcinoid syndrome and can be life-threatening. It is believed to be a result of overwhelming release of systemically acting compounds released from the tumour. Broadly, the products released are amines (e.g. Serotonin, dopamine, histamine), prostaglandins and polypeptides (e.g. Kallikreins, bradykinin) [2–4]. Carcinoid syndrome manifests as flushing, wheeze, abdominal pain and diarrhoea. In carcinoid crisis, there is an associated tachycardia and vasodilation potentially resulting in distributive shock [5]. Carcinoid crisis is typically precipitated by tumour manipulation (including biopsy), anaesthesia [6], chemotherapy or radiological procedures [7] including embolization [8].

Somatostatin analogues act as inhibitory peptides on gastrointestinal proliferative and secretory processes and are the mainstay of management to prevent and treat carcinoid syndrome and crises. However, pre-operative octreotide acetate alone is not sufficient in preventing intraoperative carcinoid crisis even in patients undergoing liver resection [5, 9]. Pre-operative imaging will not identify liver metastatic disease in 14% of patients and 75% with metastatic peritoneal disease subsequently found during surgery [10]. This suggests that any patient with a known small bowel NET should be considered for at risk of intraoperative carcinoid crisis irrespective of a diagnosis including metastatic disease.

Somatostatin analogue use is associated with impairment of gallbladder function, increasing the formation of bile crystals in the bile, inhibiting sphincter of Oddi relaxation and impairing gallbladder emptying. If not treated with somatostatin analogues, very few NEN patients (7%) suffer from symptomatic gallstones [11].

## CASE REPORT

**Figure 1:**
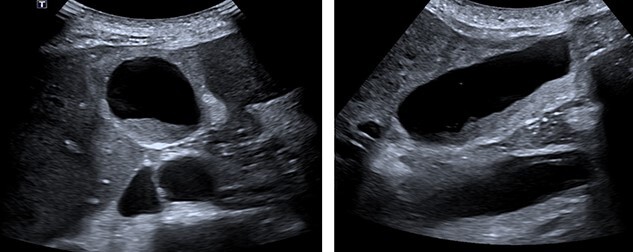
Abdominal ultrasound. A layer of mobile sludge and thickened gallbladder walls. Several liver lesions with mixed echogenicity were noted, several adjacent to the gallbladder fossa.

A 72-year-old male presented with a two-day history of right upper quadrant pain on a background of a metastatic, small bowel, well-differentiated NET diagnosed 5 years previously. The tumour was a low-grade mesenteric NET with a ki67 of 8% at diagnosis. He had multiple liver metastasis, which were stable. Carcinoid syndrome was well controlled on a long-acting, monthly dose of a somatostatin analogue with last dose being 28 days prior to presentation and due in three days. His comorbidities included hypertension, an infrarenal aortic aneurysm and chronic obstructive pulmonary disease. He presented with right upper quadrant tenderness and the liver edge was palpable 4 cm below the costal margin. Initial blood tests showed a white cell count of 18.9 × 10^9^/L, C-reactive protein 212 mg/L and liver function tests within normal limits. Vital signs were reassuring, and an abdominal ultrasound described a thickened gallbladder wall, mobile sludge and an 8.5-mm common bile duct suggestive of cholecystitis (Figure 1). Computerised tomography (CT) of the abdomen was noted to show multiple segment V liver masses with capsular deformity suggestive of central necrosis. He was initially managed with analgesia and intravenous piperacillin-tazobactam. After a discussion with the patient and consultation with his treating oncologist regarding his relatively stable disease burden and reasonable prognosis, a laparoscopic cholecystectomy was performed.

**Figure 2:**
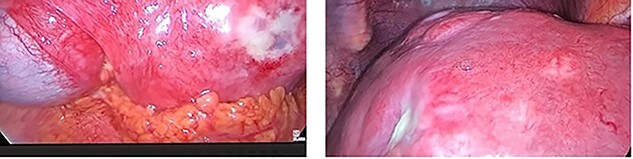
Extensive hepatic metastatic disease. An inelastic liver edge and inflamed gallbladder was encountered with adhesions.

During cholecystectomy, extensive carcinoid metastases were encountered (Figure 2). To perform the operation laparoscopically required retraction of the left liver lobe. However, the liver was stiff and inflamed making exposure of the biliary tree difficult. The gallbladder was mobilized, a critical view of safety and an unremarkable intraoperative cholangiogram was obtained. The length of the operation was 1 h and 35 min. Histopathology of the gallbladder showed benign, chronic outlet obstruction, patchy serosal mixed inflammation and one lymph node containing metastatic disease.

During the procedure, the patient developed a persistent tachycardia and an octreotide infusion was initiated. He developed hypotension, wheeze, sweating, bronchospasm and flushing. Venous pH was 7.14. Electrocardiogram showed pronounced ST-segment elevation in leads V3 and V4, multiple ventricular ectopic beats, bigeminy and bundle branch block. Troponin levels rose to >50 000 ng/L. A bedside echocardiogram showed hyperdynamic left ventricular function and septal regional wall abnormality. Profound vasodilatory and cardiogenic shock resulted requiring vasopressin at 6 mL/h (0.04 IU/min) titrated over 7 hours and noradrenaline at 5 mL/h (5ug/min) titrated over 6 h. Later, cardiac angiography showed minor coronary disease and within 24 h, he was extubated, his pain resolved, and inflammatory markers had normalised within 48 h.

## DISCUSSION

During surgical procedures, NENs are prone to pathological neuroendocrine secretion of serotonin, tachykinins, histamine, kallikreins, prostaglandin and catecholamines. Pre- and intra-operative infusion with somatostatin can mitigate the risk of on-table carcinoid crisis by reducing the release of these vasoactive mediators. The American neuroendocrine tumour society (NANETS) claims that routine administration of octreotide does not prevent a carcinoid crisis but does advise an intraoperative 100–500 μg/h infusion [12]. The European neuroendocrine society (ENETS) advises an infusion of 50–100μg/h 12 h before and 48 post-surgery [13]. The UK and Ireland neuroendocrine society (UKINETS) has developed a treatment matrix based on the type of procedure and the severity of preoperative carcinoid syndrome symptoms [14]. The decision is often made at the discretion of the anaesthetist. In high acuity surgical scenarios, optimization of measures to prevent carcinoid crisis may be unattainable.

Hypotension is the most common problem during anaesthesia, and there is a theoretical risk of worsening hypotension by triggering further release of vasoactive peptides [13]. However, this view is not widely accepted as several reports have shown that the use of inotropic agents along with octreotide can restore cardiovascular stability [15]. This response was observed in the case where hypotension responded to catecholamine infusions.

To date, there are no cases reported of carcinoid crisis at the time of laparoscopic cholecystectomy for cholecystitis. Performing the surgery required manipulation of the liver, which likely released vasoactive mediators precipitating the crisis. The crisis could, however, be explained by anaesthesia and although the CT demonstrated necrotic metastasis, the patient was previously asymptomatic making necrosis alone a less likely explanation.

Treatment with somatostatin analogues is known to cause biliary stasis, which can sometimes become symptomatic and require surgical management. The patient in this case carried an increased risk of developing gallstones and subsequently suffering cholecystitis due to somatostatin analogue use. This case supports the practice of prophylactic cholecystectomy [11] in this patient group undergoing abdominal surgery for other reasons such as debulking or bowel resections.
